# 5,8-Dihydroxy-4 ′, 7-dimethoxyflavone Attenuates TNF-*α*-Induced Expression of Vascular Cell Adhesion Molecule-1 through EGFR/PKC*α*/PI3K/Akt/Sp1-Dependent Induction of Heme Oxygenase-1 in Human Cardiac Fibroblasts

**DOI:** 10.1155/2022/1372958

**Published:** 2022-03-04

**Authors:** Chien-Chung Yang, Li-Der Hsiao, Ya-Fang Shih, Hsin-Hui Lin, Chuen-Mao Yang

**Affiliations:** ^1^Department of Traditional Chinese Medicine, Chang Gung Memorial Hospital at Tao-Yuan, Kwei-San, Tao-Yuan 33302, Taiwan; ^2^School of Traditional Chinese Medicine, College of Medicine, Chang Gung University, Kwei-San, Tao-Yuan 33302, Taiwan; ^3^Department of Pharmacology, College of Medicine, China Medical University, Taichung 40402, Taiwan; ^4^Ph.D. Program for Biotech Pharmaceutical Industry, China Medical University, Taichung 40402, Taiwan; ^5^Department of Post-Baccalaureate Veterinary Medicine, College of Medical and Health Science, Asia University, Wufeng, Taichung 41354, Taiwan

## Abstract

Recently, we found that 5,8-dihydroxy-4′,7-dimethoxyflavone (DDF) upregulated the expression of heme oxygenase (HO)-1 via p38 mitogen-activated protein kinase/nuclear factor-erythroid factor 2-related factor 2 (MAPK/Nrf2) pathway in human cardiac fibroblasts (HCFs). However, the alternative processes by which DDF induces the upregulation of HO-1 expression are unknown. Activation of epidermal growth factor receptor (EGFR), phosphoinositide 3-kinase/protein kinase B (PI3K/Akt), and protein kinase C (PKC)*α* may initiate specificity protein (Sp)1 activity, which has been reported to induce expression of antioxidant molecules. Thus, we explored whether these components are engaged in DDF-induced HO-1 upregulation in HCFs. Western blotting, promoter-reporter analyses, and real-time polymerase chain reactions were adopted to measure HO-1 and vascular cell adhesion molecule (VCAM)-1 expressions in HCFs. Respective small interfering (si)RNAs and pharmacological inhibitors were employed to investigate the signaling components engaged in DDF-induced HO-1 upregulation. The chromatin immunoprecipitation assay was conducted to detect the binding interaction of Sp1 and antioxidant response elements (ARE) on the promoter of HO-1. An adhesion assay of THP-1 monocyte was undertaken to examine the functional effect of HO-1 on tumor necrosis factor (TNF)-*α*-induced VCAM-1 expression. DDF stimulated the EGFR/PKC*α*/PI3K/Akt pathway leading to activation of Sp1 in HCFs. The roles of these protein kinases in HO-1 induction were ensured by transfection with their respective siRNAs. Chromatin immunoprecipitation assays revealed the interaction between Sp1 and the binding site of proximal ARE on the HO-1 promoter, which was abolished by glutathione, AG1478, Gö6976, LY294002, or mithramycin A. HO-1 expression enhanced by DDF abolished the monocyte adherence to HCFs and VCAM-1 expression induced by TNF-*α*. Pretreatment with an inhibitor of HO-1: zinc protoporphyrin IX reversed these inhibitory effects of HO-1. We concluded that DDF-induced HO-1 expression was mediated via an EGFR/PKC*α*/PI3K/Akt-dependent Sp1 pathway and attenuated the responses of inflammation in HCFs.

## 1. Introduction

Fibroblasts are crucial components in the pathogenesis of hearts (e.g., cardiac inflammation) induced by chemical signals or mechanical forces. Proinflammatory cytokines and mediators, such as tumor necrosis factor (TNF)-*α*, interleukin (IL)-1*β*, IL-6, and sphingosine 1-phosphate, promote the activation and proliferation of fibroblasts [1–3]. For example, TNF-*α* overexpression-induced fibrotic cardiomyopathy and interactions between mast cells and fibroblasts are needed for the progress of cardiac fibrosis [4]. Turner et al. [5] suggested that TNF-*α* increases the levels of IL-1*β* and IL-6 in cardiac fibroblasts. Our recent report indicated that TNF-*α* and sphingosine 1-phosphate induce cyclooxygenase-2/prostaglandin E_2_ upregulation in human cardiac fibroblasts (HCFs) [3, 6]. Those findings imply that TNF-*α* is a crucial factor in cardiovascular disorders.

The adhesion of inflammatory cells to the vascular endothelium is mediated through vascular cell adhesion molecule (VCAM)-1 during the processes of inflammation. Savic-Radojevic et al. [7] found that the prevalence of mortality and morbidity in chronic heart failure patients could be predicted by the levels of VCAM-1 and TNF-*α*. Previously, we revealed that upregulation of VCAM-1 expression can be enhanced in response to TNF-*α* in HCFs [8]. These proinflammatory mediators, through upregulation of VCAM-1 expression, may be implicated in cardiac inflammation. Therefore, these components could be targets for developing an antioxidant strategy to provide an efficacious intervention in cardiovascular diseases.

Evidence suggests that stimuli- and oxidative stress-induced inflammation could be protected by heme oxygenase (HO)-1 [9, 10]. Thus, HO-1 has been considered an efficacious therapeutic intervention for managing various human diseases, including cardiac inflammation. For HO-1 inducers, considerable attention has been focused on Chinese herbal medicines used in the treatment of inflammatory diseases. Cardenolides extracted from the root of *Reevesia formosana* have been shown to exhibit potent cytotoxicity in cancer cell lines *in vitro* [11]. Flavonoids have demonstrated their efficacy via antioxidant and anti-inflammatory effects in several pathological conditions, including cardiovascular diseases. The flavonoid 5,8-dihydroxy-4′,7-dimethoxyflavone (DDF) is another component isolated from *R. formosana* [12]. In HCFs, DDF has been shown to stimulate the expression of HO-1 (at least in part) via nuclear factor-erythroid factor 2-related factor 2 (Nrf2) activation dependent on p38 mitogen-activated protein kinase (MAPK) and reactive oxygen species (ROS) pathways [12]. However, how DDF promotes the expression of HO-1 and exerts cytoprotective effects in HCFs is not known.

Various signaling pathways have been demonstrated to modulate the upregulation of HO-1 expression induced by different stimuli [13]. For instance, puerarin modulates HO-1 induction via a protein kinase C- (PKC-) dependent pathway in mouse mesangial cells [14]. In A549 cells, HO-1 induction by cisplatin is mediated via phosphoinositide 3-kinase/protein kinase B (PI3K/Akt) pathways dependent on epidermal growth factor receptor (EGFR) activation [15]. Moreover, several transcriptional factors [e.g., Nrf2, activating-protein (AP)1, specificity protein (Sp)1] are controlled by various signaling pathways, which participate in *HO-1* expression [16, 17]. Here, we explored the molecular mechanisms underlying DDF-induced expression of HO-1 and “rescues” TNF-*α*-stimulated proinflammatory reactions in HCFs.

## 2. Material and Methods

### 2.1. Chemicals, Antibodies, and Reagents

Hybond C membrane and reagents for enhanced chemiluminescence (ECL), fetal bovine serum (FBS), Dulbecco's modified Eagle's medium (DMEM)/F-12, and sodium dodecyl sulfate–polyacrylamide gel electrophoresis (SDS–PAGE) were used. This study used the method of Yang et al. [12]. Antibodies against phosphorylated (phospho)-EGFR (Tyr^1068^; catalog number, 2234) and phospho-Akt (Ser^473^; 9271) were purchased from Cell Signaling Technologies (Danvers, MA, USA). Antibody against phospho-Sp1 (Thr^453^; A0577) was obtained from Assay Biotech (Sunnyvale, CA, USA). Antibodies against phospho-PKC*α* (phospho-S^657^; EPR1901(2); ab180848) and VCAM-1 (EPR5038(2); ab174279) were purchased from Abcam (Cambridge, UK). Antibodies against Sp1 (sc-14027), PKC*α* (C20; sc-208), Akt (sc-8312), EGFR (1005; sc-03), and anti-*β*-actin (C4; sc-47778) were from Santa Cruz Biotechnology (Santa Cruz, CA, USA). AG1478, Gö6976, LY294002, glutathione (GSH), and mithramycin A were sourced from Biomol (Plymouth Meeting, PA, USA). Anti-glyceraldehyde 3-phosphate dehydrogenase (GAPDH; MCA-1D4) was purchased from EnCor (Gainesville, FL, USA). Anti-HO-1 polyclonal antibody (ADI-SPA-895) was obtained from Enzo Life Sciences (Farmingdale, NY, USA). Zinc protoporphyrin (ZnPP) IX was from Cayman Chemicals (Ann Arbor, MI, USA). Recombinant human TNF-*α* protein was sourced from R&D Systems (Minneapolis, MN, USA). Other chemicals and enzymes were purchased from MilliporeSigma (Burlington, MA, USA).

### 2.2. Culture and Treatment of Cells

HCFs were purchased from ScienCell Research Laboratories (San Diego, CA, USA). HCFs were cultured and treated using the method of Yang et al. [12]. HCFs were cultured at 37°C in a humidified atmosphere of 5% CO_2_ in DMEM/F-12 supplemented with 10% FBS and antibiotics. When the cultures reached confluence (~4 days), the cells were suspended by 0.05% trypsin/0.53 mM EDTA and diluted with DMEM/F-12 containing 10% FBS to a concentration of 2 × 10^5^ cells/ml. The cell suspension was seeded onto 10 cm culture dishes (10 ml/dish), six-well culture plates (2 ml/well), and 12-well culture plates(1 ml/well). The cells were made quiescent by incubation in serum-free DMEM/F-12 for 24 h, pretreated with the inhibitors for 1 h, and then incubated with DDF at 37°C for the time intervals indicated. HCF passages from 5 to 7 were used for subsequent experiments.

### 2.3. Preparation of Samples and Western Blotting

The samples were prepared and analyzed by western blotting using the method of Yang et al. [12]. Growth-arrested cells by incubation in serum-free DMEM/F-12 for 24 h were pretreated with inhibitors for 1 h and then incubated with DDF at 37°C for the time intervals indicated. After incubation, the cells were washed rapidly with ice-cold phosphate-buffered saline (PBS) and lysed with sample buffer. Proteins were separated by SDS–PAGE and transferred by electrophoresis onto nitrocellulose membranes (BioTrace™ NT membranes; Pall Life Sciences, Ann Arbor, MI, USA). Nitrocellulose membranes were incubated sequentially with a specific primary antibody overnight, followed by incubation with a secondary horseradish peroxidase-conjugated antibody for 1 h. An internal control either an anti-*β*-actin antibody or anti-GAPDH antibody was used for protein loading. Immunoreactive bands on nitrocellulose membranes were detected using ECL reagents and captured by the BioSpectrum™ 500 Imaging System (Ultra-Violet Products, Upland, CA, USA). To quantify image densitometry, UN-SCAN-IT gel software (Silk Scientific, Orem, UT, USA) was used.

### 2.4. Extraction of Total RNA and Real-Time Reverse Transcription-Quantitative Polymerase Chain Reaction (RT-qPCR)

HCFs were treated with DDF for the time intervals indicated in the absence or presence of inhibitors. Total RNA was extracted for real-time RT-qPCR using the method of Yang et al. [12]. Total RNA was extracted with TRIzol® reagent from HCFs. Synthesis of first-strand complementary (c)DNA was done for 60 min at 37°C with 2 *μ*g of total RNA using random hexamers as primers in a final volume of 20 *μ*l, as described previously. The synthesized cDNA molecules were used as templates for PCR using primers for target genes and Q-Amp™ 2× screening Fire Taq Master Mix (Bio-Genesis Technologies, Taipei, Taiwan). qPCR was done using the Kapa Probe Fast qPCR Kit Master Mix Universal (Kapa Biosystems, Wilmington, MA, USA) on a StepOnePlus™ real-time PCR system (Thermo Scientific, Waltham, MA, USA). To calculate the relative amount of the target genes, the *^ΔΔ^*Ct method (Ct = threshold cycle) was used. The primer sequences (forward and reverse, respectively) were 5′-CTCCCAGGCTCCGCTTCT-3′ and 5′-GCATGCCTGCATTCACATG-3′ for HO-1 and 5′-GCCAGCCGAGCCACAT-3′ and 5′-CTTTACCAGAGTTAAAAGCAGCCC-3′ for GAPDH.

### 2.5. Transient Transfection with Small Interfering (si)RNAs

The procedures for siRNA transfection were adopted from the method of Yang et al. [12]. HCFs of 2 × 10^5^ cells/ml were plated onto 12-well plates or 10 cm dishes until ~70% confluence was reached. Before transfection, the cells were washed with PBS and then added to 1 ml/well or 5 ml/dish of Opti-MEM medium (Gibco, Grand Island, NY, USA). Akt siRNA (SASI_Hs01_00105954), PKC*α* siRNA (SASI_Hs01_00018816), and Sp1 siRNA (SASI_Mm01_00145222) were obtained from MilliporeSigma. EGFR siRNA (sense: 5′-GAAGGAAA CUGAAUUCAAA-3′ and antisense: 5′-UUUGAAUUCAGUUUCCUUC-3′) was purchased from MDBio (Taipei, Taiwan). Lipofectamine™ 2000 transfection reagent (Invitrogen, Carlsbad, CA, USA) was used to carry out transient transfection of siRNAs. Complexes of DNA–Lipofectamine transfection reagent were added to each well to a final concentration of 100 nM siRNA, and then, incubation for 5 h at 37°C was carried out. After transfection, the cells were made quiescent and then treated with DDF.

### 2.6. Transfection and Promoter Luciferase Assay

We investigated the effect of TNF-*α* on VCAM-1 activity. A VCAM-1-luc plasmid was constructed, and luciferase activity was analyzed using the method of Lee et al. [18]. A region spanning −1716 bp to −119 bp for the human VCAM-1 promoter, which was kindly provided by Dr. W.C. Aird (Department of Molecular Medicine, Beth Israel Deaconess Medical Center, Boston, MA, USA), was cloned into a pGL3-basic vector (Promega, Madison, WI, USA). The VCAM-1-luc reporter gene was transfected transiently, and the control pGal encoding for *β*-galactosidase was present to normalize for transfection efficiency. A luciferase assay system (Promega) was adopted to analyze the luciferase activity. The luciferase activity of the firefly was standardized for *β*-galactosidase activity.

### 2.7. Chromatin Immunoprecipitation (ChIP) Assay

To detect the association of Sp1 with the promoter of human HO-1, the ChIP assay was undertaken using the method of Yang et al. [12]. HCFs in 10 cm dishes were grown in a serum-free environment for 24 h to reach confluence and then treated with DDF. One percent of formaldehyde in the medium was added to fix protein–DNA complexes. Fixed cells were washed and lysed in an SDS-lysis buffer. Cell lysates kept at 4°C were sonicated until the DNA size was 200–300 bp. The samples were centrifuged, and soluble chromatin was precleared by incubation with sheared salmon-sperm DNA–protein agarose A for 30 min at 4°C with rotation.

After preclearing, samples were centrifuged, and the supernatant was transferred to a new tube. The concentrations of samples were quantified and adjusted. One portion of the sample was used as a DNA input control, and the remainder was incubated with anti-Sp1 antibody overnight at 4°C. Protein A beads (MilliporeSigma) were added overnight with rotation at 4°C to collect the immunoprecipitating complexes of antibody–protein–DNA. After incubation, the samples were sequentially washed with low-salt buffer, high-salt buffer, LiCl buffer, and Tris-EDTA and then eluted with elution buffer, as described previously. To extract DNA, the crosslinking of protein–DNA complexes were reversed by incubation at 65°C overnight. The extracted DNA was resuspended in H_2_O and subjected to PCR amplification using the TaqMan™ ChIP QPCR Assay (ThermoScientific).

### 2.8. Adhesion Assay

HCFs plated onto six-well plates were grown to confluence and incubated with TNF-*α* for 16 h, and then, adhesion assays were undertaken using the method of Lee et al. [18]. Briefly, THP-1 cells (human acute monocytic cell line) were labeled with the fluorescent dye 2′,7′-bis-(2-carboxyethyl)-5-(and-6)-carboxyfluorescein, acetoxymethyl ester (20 *μ*M) for 1 h in PBS at 37°C and washed subsequently by centrifugation. HCFs stimulated by TNF-*α* for 16 h were incubated with THP-1 cells for 1 h at 37°C. HCFs were washed gently thrice with PBS to remove nonadherent THP-1 cells. To determine the number of adherent THP-1 cells, the cells were counted by five fields per 20× field well using a fluorescence microscope (Axiovert 200 M; Carl Zeiss, Thornwood, NY, USA).

### 2.9. Statistical Analyses

To determine the statistical analysis, Prism 6.0 (GraphPad, San Diego, CA, USA) was employed. The methods of statistical analyses were adopted from the method of Yang et al. [12]. We used one-way ANOVA followed by Dunnett's post hoc test if comparing more than two groups of data, as described previously, or the nonparametric Kruskal–Wallis test followed by Dunn's multiple comparison test if comparing multiple independent groups and if the assumptions of ANOVA normality were not met. *Post hoc* tests were run only if F achieved *p* < 0.01 and there was no significant variance in homogeneity. Data are the mean ± SEM. *p* < 0.01 was considered significant.

## 3. Results

### 3.1. DDF Induces Upregulation of the Expression of HO-1 via EGFR in HCFs

Our recent report has shown that ROS generation via GSH depletion mediates upregulated expression of HO-1 induced by DDF in HCFs [12]. ROS can be second messengers and activate their downstream signaling components such as platelet-derived growth factor receptor (PDGFR) and EGFR in various cell types, which leads to increased levels of HO-1 [19, 20]. Therefore, we examined the role of EGFR on DDF-induced responses.

**Figure 1:**
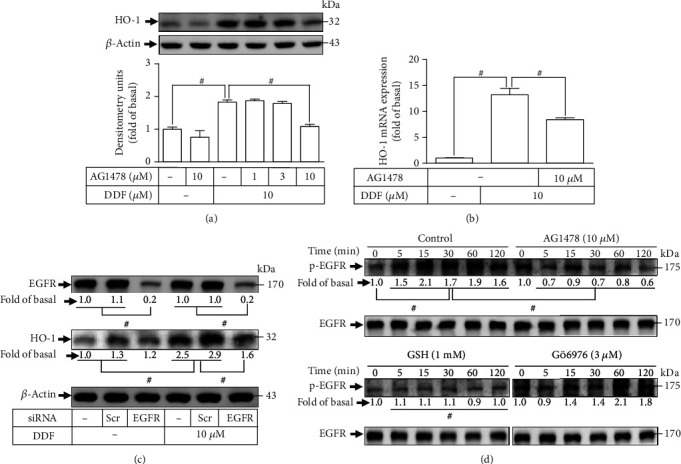
DDF induces HO-1 expression via ROS/EGFR in HCFs. (a) HCFs were pretreated with AG1478 for 1 h and then incubated with DDF (10 *μ*M) for 16 h. The levels of HO-1 and *β*-actin protein expressions were examined by western blot analysis. (b) Cells were pretreated with AG1478 for 1 h and then incubated with DDF (10 *μ*M) for 6 h. The levels of HO-1 mRNA were analyzed by real-time PCR. (c) Cells were transfected with scrambled or EGFR siRNA and then incubated with DDF (10 *μ*M) for 16 h. The levels of EGFR, HO-1, and *β*-actin protein expression were determined by western blot analysis. (d) Cells were pretreated without or with GSH, Gö6976, or AG1478 and then incubated with DDF (10 *μ*M) for the indicated time intervals. The levels of phospho- and total-EGFR were determined by western blot. In this part, to determine the effect of AG1478, GSH, or Gö6976 on DDF-stimulated phosphorylation of EGFR, these experiments were conducted in the absence (control) or presence of inhibitor and then incubated with DDF for the indicated time intervals. To fit the construct of data layout, only one set of control was presented and disclosed by the insertion of white spaces rearranged from the original capture. Data are expressed as mean ± SEM of three independent experiments (*n* = 3). ^#^*p* < 0.01, as compared with DDF alone. Abbreviations: Scr: scramble.

To investigate the role of EGFR, AG1478 (EGFR inhibitor) was adopted, and the dose and time interval of DDF treatment were based on our recent report [12]. AG1478 pretreatment dose-dependently reduced the expression of HO-1 produced by DDF (Figure 1(a)). AG1478 diminished HO-1 transcription induced by DDF as well (Figure 1(b)). EGFR siRNA was transfected into HCFs to knockdown EGFR expression. Downregulation of EGFR expression attenuated DDF-enhanced protein level of HO-1 **(**Figure 1(c)). Moreover, phospho-EGFR expression was measured using western blotting to determine the role of EGFR phosphorylation in DDF-triggered upregulation of HO-1 expression. EGFR phosphorylation was time-dependently stimulated by DDF (Figure 1(d)). Moreover, pretreatment with GSH or AG1478 abrogated the EGFR phosphorylation stimulated by DDF in HCFs, but pretreatment with Gö6976 did not. These findings from HCFs suggested that activation of ROS/EGFR modulates DDF-induced HO-1 induction.

### 3.2. Involvement of PKCs in Upregulation of HO-1 Expression Induced by DDF

PKCs are involved in several cellular functions and the pathogenesis of various diseases in which redox-sensitive signaling molecules are involved [21]. PKC activation in mouse mesangial cells has been shown to suppress inflammation related to advanced glycation end products via increased expression of HO-1 [14]. Thus, we assessed the effects of PKCs on the DDF-induced expression of HO-1.

**Figure 2:**
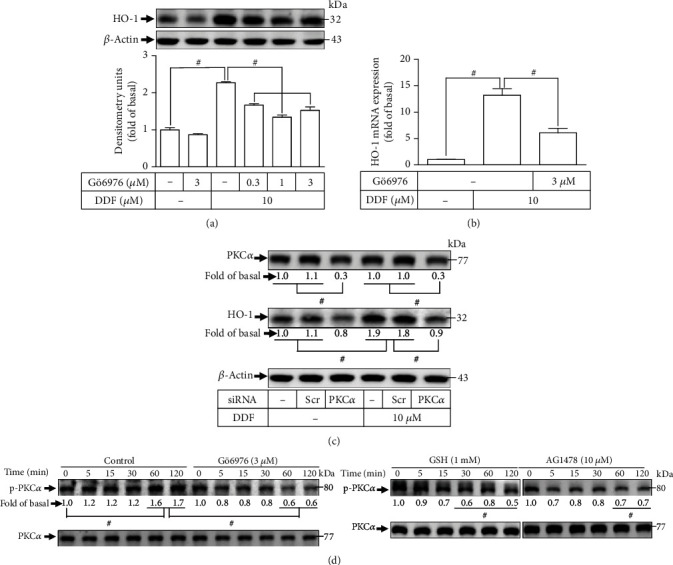
Involvement of PKC*α* in DDF-induced HO-1 expression. (a) HCFs were pretreated with Gӧ6976 for 1 h and then incubated with DDF (10 *μ*M) for 16 h. The levels of HO-1 and *β*-actin protein expressions were examined by western blot analysis. (b) Cells were pretreated with Gӧ6976 for 1 h and then incubated with DDF (10 *μ*M) for 6 h. The levels of HO-1 mRNA were analyzed by real-time PCR. (c) Cells were transfected with scrambled or PKC*α* siRNA and then incubated with DDF (10 *μ*M) for 16 h. The levels of PKC*α*, HO-1, and *β*-actin protein expressions were determined by western blot analysis. (d) Cells were pretreated without or with Gö6976, GSH, or AG1478 and then incubated with DDF (10 *μ*M) for the indicated time intervals. The levels of phospho- and total-PKC*α* were determined by western blot. In this part, to determine the effect of Gö6976, GSH, or AG1478 on DDF-stimulated phosphorylation of PKC*α*, these experiments were conducted in the absence (control) or presence of inhibitor and then incubated with DDF for the indicated time intervals. To fit the construct of data layout, only one set of control was presented and disclosed by the insertion of white spaces rearranged from the original capture. Data are expressed as mean ± SEM of three independent experiments (*n* = 3). ^#^*p* < 0.01, as compared with DDF alone.

To test this hypothesis, Gö6976 (selective inhibitor of PKC*α*) was used. Gö6976 dose-dependently blocked the DDF-enhanced increased protein expression of HO-1 (Figure 2(a)) and transcription (Figure 2(b)) in HCFs. PKC*α* siRNA was adopted to ascertain the role of PKC*α*. Downregulation of expression of PKC*α* protein using transfection of PKC*α* siRNA diminished DDF-induced upregulation of HO-1 (Figure 2(c)). Moreover, we investigated if PKC*α* phosphorylation participated in the upregulation of HO-1 induced by DDF using western blotting. Gö6976 pretreatment reduced DDF-stimulated time-dependent PKC*α* phosphorylation (Figure 2(d)). In addition, in HCFs, pretreatment with GSH or AG1478 attenuated DDF-stimulated PKC*α* phosphorylation. These results in HCFs indicated that the ROS/EGFR/PKC*α* pathway modulates upregulation of HO-1 generated by DDF.

### 3.3. DDF Enhances Upregulation of HO-1 through PI3K/Akt in HCFs

**Figure 3:**
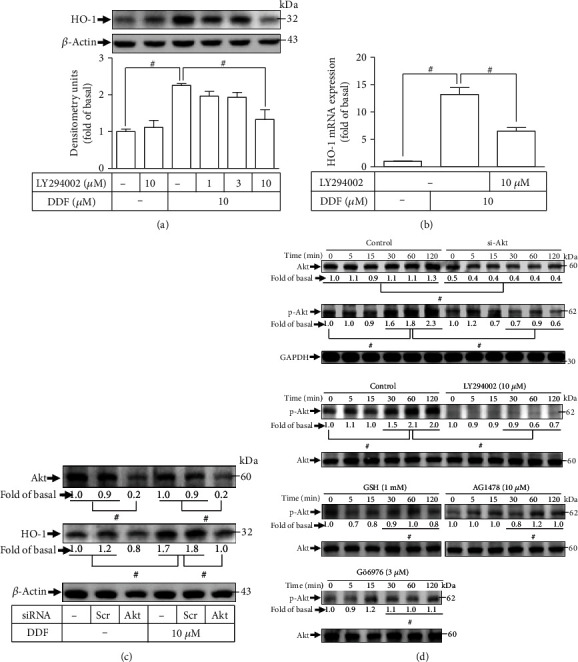
DDF induces HO-1 expression via PI3K/Akt in HCFs. (a) HCFs were pretreated with LY294002 for 1 h and then incubated with DDF (10 *μ*M) for 16 h. The levels of HO-1 and *β*-actin protein expressions were examined by western blot analysis. (b) Cells were pretreated with LY294002 for 1 h and then incubated with DDF (10 *μ*M) for 6 h. The levels of HO-1 mRNA were analyzed by real-time PCR. (c) Cells were transfected with scrambled or Akt siRNA and then incubated with DDF (10 *μ*M) for 16 h. The levels of Akt, HO-1, and *β*-actin protein expressions were determined by western blot analysis. (d) Cells were transfected with Akt siRNA or pretreated with GSH, AG1478, LY294002, or Gö6976 for 1 h, and then incubated with DDF (10 *μ*M) for the indicated time intervals. The levels of phospho- and total-Akt were determined by western blot. In this part, to determine the effect of LY294002, GSH, AG1478, or Gö6976 on DDF-stimulated phosphorylation of Akt, these experiments were conducted in the absence (control) or presence of inhibitor and then incubated with DDF for the indicated time intervals. To fit the construct of data layout, only one set of control was presented and disclosed by the insertion of white spaces rearranged from the original capture. Data are expressed as mean ± SEM of three independent experiments (*n* = 3). ^#^*p* < 0.01, as compared with DDF alone. Abbreviations: si-Akt: siRNA of Akt.

Upregulation of HO-1 expression can result from PI3K/Akt in several cell types [22]. EGFR has been elucidated to be an upstream component of PI3K/Akt, inducing the expression of HO-1 in cells [20, 23, 24]. Previously, using mouse brain endothelial cells, we revealed that HO-1 induction is promoted by cigarette smoke extract via PKC to activate the downstream components PI3K/Akt [25]. Thus, we assessed the effect of PI3K/Akt on DDF-stimulated responses. HCFs were pretreated with a PI3K inhibitor (LY294002) or transfected with Akt siRNA. As shown in Figure 3(a), pretreatment with LY294002 dose-dependently reduced DDF-enhanced expression of HO-1 protein. Moreover, LY294002 suppressed the expression of HO-1 mRNA induced by DDF (Figure 3(b)). Akt expression knocked down by Akt siRNA was used to verify the effect of Akt on DDF-induced HO-1 expression. Downregulation of expression of Akt protein by Akt siRNA transfection impeded DDF-induced HO-1 upregulation (Figure 3(c)). Moreover, we investigated (by western blotting) Akt phosphorylation involved in the DDF-induced responses. In HCFs, Akt siRNA transfection or LY294002 pretreatment attenuated DDF-stimulated Akt phosphorylation (Figure 3(d)). Pretreatment with GSH, AG1478, or Gö6976 attenuated DDF-stimulated Akt phosphorylation (Figure 3(d)). These results in HCFs revealed that activation of a ROS/EGFR/PKC*α*-dependent PI3K/Akt cascade modulates DDF-induced upregulation of HO-1 expression.

### 3.4. The Transcription Factor Sp1 Engages in DDF-Induced Increased Expression of HO-1

**Figure 4:**
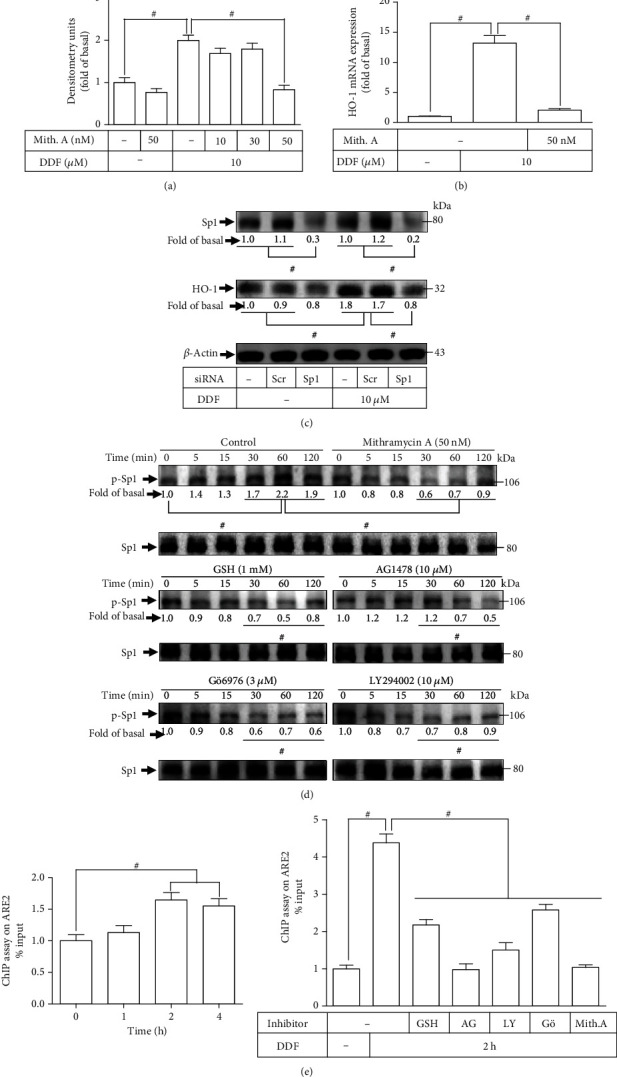
Transcription factor Sp1 is involved in DDF-induced HO-1 expression. (a) HCFs were pretreated with mithramycin A for 1 h and then incubated with DDF (10 *μ*M) for 16 h. The levels of HO-1 and *β*-actin protein expressions were examined by western blot analysis. (b) Cells were pretreated with mithramycin A for 1 h and then incubated with DDF (10 *μ*M) for 6 h. The levels of HO-1 mRNA were analyzed by real-time PCR. (c) Cells were transfected with scrambled or Sp1 siRNA and then incubated with DDF (10 *μ*M) for 16 h. The levels of Sp1, HO-1, and *β*-actin protein expressions were determined by western blot analysis. (d) Cells were pretreated without or with GSH, AG1478, LY294002, Gö6976, or mithramycin A for 1 h and then incubated with DDF (10 *μ*M) for the indicated time intervals. The levels of phospho- and total-Sp1 were determined by western blot. In this part, to determine the effect of mithramycin A, GSH, AG1478, LY294002, Gö6976, or LY294002 on DDF-stimulated phosphorylation of Sp1, these experiments were conducted in the absence (control) or presence of inhibitor and then incubated with DDF for the indicated time intervals. To fit the construct of data layout, only one set of control was presented and disclosed by the insertion of white spaces rearranged from the original capture. (e) Cells were incubated with DDF for the indicated time intervals (left panel). Cells were preincubated with GSH, AG1478, LY294002, Gö6976, or mithramycin A for 1 h and then incubated with DDF for 2 h (right panel). The transcriptional activity of Sp1 was analyzed by a ChIP assay. Data are expressed as mean ± SEM of three independent experiments (*n* = 3). ^#^*p* < 0.01, as compared with DDF alone. Abbreviations: Mith.A: mithramycin A; AG: AG1478; LY: LY294002; Gö: Gö6976.

The Sp1 has been demonstrated to cooperate with Nrf2 to induce HO-1 expression stimulated by carbon monoxide-releasing molecule (CORM)-2 [26]. Previously, in RBA-1 cells, we found that PI3K/Akt activates recruitment of the complex of Sp1, Nrf2, and c-Jun, which results in HO-1 induction [26]. We wished to investigate if Sp1 participated in HO-1 expression induced by DDF. Pretreatment of cells with mithramycin A (an Sp1 inhibitor) dose-dependently reduced the protein levels of HO-1 enhanced by DDF (Figure 4(a)). Pretreatment with mithramycin A also reduced the DDF-induced mRNA expression of HO-1 (Figure 4(b)). Transfection with Sp1 siRNA was used to ascertain the role of Sp1 on DDF-induced upregulation of HO-1. As shown in Figure 4(c), downregulation of the expression of Sp1 protein by transfection with Sp1 siRNA diminished DDF-induced HO-1 expression. Sp1 phosphorylation was measured by western blotting to determine how phosphorylation of Sp1 regulated the upregulated level of HO-1 by DDF. Pretreatment with mithramycin A attenuated phosphorylation of Sp1 time-dependently stimulated by DDF (Figure 4(d)). DDF-stimulated Sp1 phosphorylation was also mitigated by pretreatment with GSH, AG1478, LY294002, or Gö6976.

Previously, we revealed that in RBA-1 cells, CORM-2 activates Sp1 and promotes its interaction with the binding site of antioxidant response elements (ARE), which results in HO-1 expression [26]. The ChIP assay was conducted to investigate if DDF-stimulated Sp1 phosphorylation was involved in the interaction with the binding site of ARE. DDF-stimulated Sp1 phosphorylation time-dependently enhanced its association with the binding site of ARE, which was diminished by pretreatment with GSH, AG1478, Gö6976, LY294002, or mithramycin A (Figure 4(e)). These data suggested that in HCFs, the ROS/EGFR/PKC*α*/PI3K/Akt cascade participates in the DDF-stimulated phosphorylation of Sp1, which leads to HO-1 expression.

### 3.5. DDF Attenuates the Effect of TNF-*α* on VCAM-1 Induction

**Figure 5:**
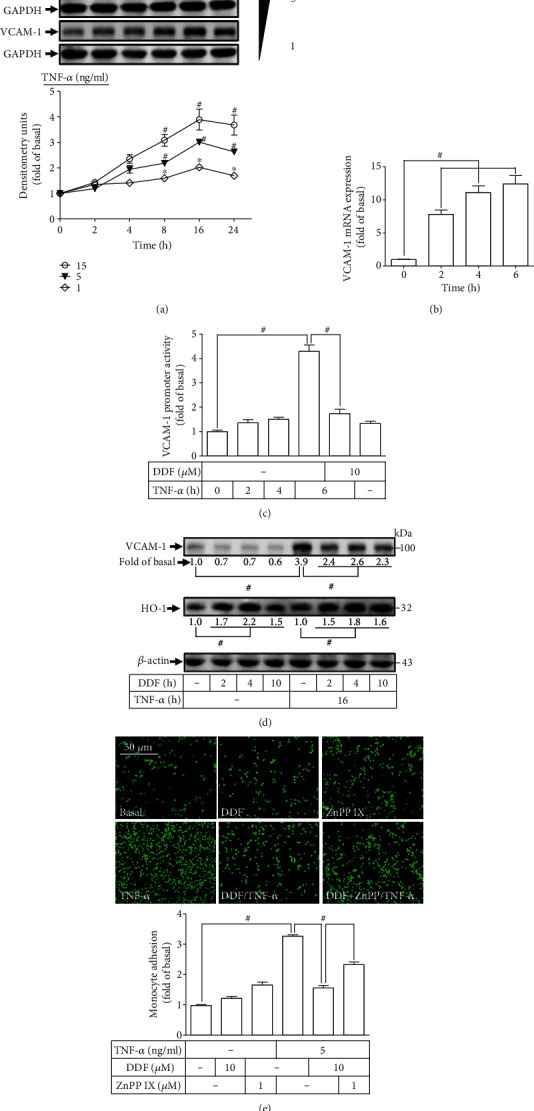
DDF protects against TNF-*α*-mediated VCAM-1 expression. (a) HCFs were incubated with various concentrations of TNF-*α* (1, 5, and 15 ng/ml) for the indicated time intervals (0, 2, 4, 8, 16, and 24 h). The levels of VCAM-1 and GAPDH protein expressions were examined by western blot analysis. (b) Cells were incubated with TNF-*α* (5 ng/ml) for the indicated time intervals (0, 2, 4, and 6 h). The levels of VCAM-1 mRNA expression were determined by real-time PCR. (c) The cells were cotransfected with VCAM-1 promoter-Luc and *β*-galactosidase and then incubated with TNF-*α* (5 ng/ml) for 0, 2, 4, or 6 h in the absence or presence of DDF (10 *μ*M). The cell lysates were used to determine the VCAM-1 promoter luciferase activity. (d) Cells were pretreated with DDF (10 *μ*M) as the indicated time intervals (0, 2, 4, and 10 h) and challenged with TNF (5 ng/ml) for 16 h. The levels of VCAM-1, HO-1, and *β*-actin protein were examined by western blot analysis. (e) Cells were pretreated with or without ZnPP IX (1 *μ*M) for 1 h and DDF (10 *μ*M) for 10 h and then incubated with TNF-*α* (5 ng/ml) for 16 h. The THP-1 cell adherence was measured using a fluorescence microscope (scale bars = 50 *μ*m). Data are expressed as mean ± SEM of three independent experiments (*n* = 3). ^#^*p* < 0.01 and ^∗^*p* < 0.05 as compared with control or TNF-*α* alone. Abbreviations: ZnPP IX: zinc protoporphyrin IX.

Previously, we found that TNF-*α* triggers inflammation in HCFs through induction of VCAM-1 and COX-2 expressions [6, 27]. Moreover, the interplay between TNF-*α* and VCAM-1 plays an important part in cardiovascular disorders [7, 28]. Thus, we investigated if DDF protected against VCAM-1 induction by the effect of TNF-*α* in HCFs. First, we evaluated the expression of VCAM-1 protein and *VCAM-1* gene by the effect of TNF-*α*. TNF-*α* time- and dose-dependently induced protein levels of VCAM-1, upregulated significantly within 4 h and achieved the maximum of expression within 16 h (Figure 5(a)). TNF-*α* (5 ng/ml) induced mRNA expression of VCAM-1 in a time-dependent manner and reached maximal expression within 6 h in HCFs (Figure 5(b)). We wished to ascertain if TNF-*α* through regulation of mRNA transcriptional activity exerted its effect on VCAM-1 induction. Hence, the promoter activity of VCAM-1 was investigated using a firefly luciferase gene reporter. TNF-*α* time-dependently stimulated the promoter activity of VCAM-1 within 6 h, and this effect was inhibited by DDF (Figure 5(c)). Furthermore, to ascertain if DDF could diminish TNF-*α*-stimulated protein expression of VCAM-1, HCFs were pretreated with DDF (10 *μ*M) for the time points indicated and subsequently challenged with 5 ng/ml TNF-*α* for 16 h. Increased expression of HO-1 in HCFs by DDF significantly inhibited the expression of VCAM-1 protein enhanced by TNF-*α* at these time points tested (Figure 5(d)). Taken together, these data suggested that in HCFs, DDF attenuates the upregulated expression of VCAM-1 enhanced by TNF-*α* through interfering with transcriptional activity by increased expression of HO-1.

To further examine the cellular function of upregulated expression of VCAM-1 stimulated by TNF-*α*, our experiments measured the adhesion of THP-1 monocytes. HCFs were treated by the presence or absence of DDF (10 *μ*M) for 10 h or together with ZnPP IX (1 *μ*M) for 1 h and then challenged (or not challenged) with TNF-*α* for 16 h. As shown in Figure 5(e), preincubation with DDF diminished the adhesion of THP-1 monocytes to HCFs stimulated by TNF-*α*, and this effect was reversed by pretreating cells with ZnPP IX. The above findings demonstrated that upregulated expression of HO-1 by DDF could protect against monocyte adhesion for HCFs exposed to TNF-*α*-enhanced upregulation of VCAM-1 expression.

## 4. Discussion

**Figure 6:**
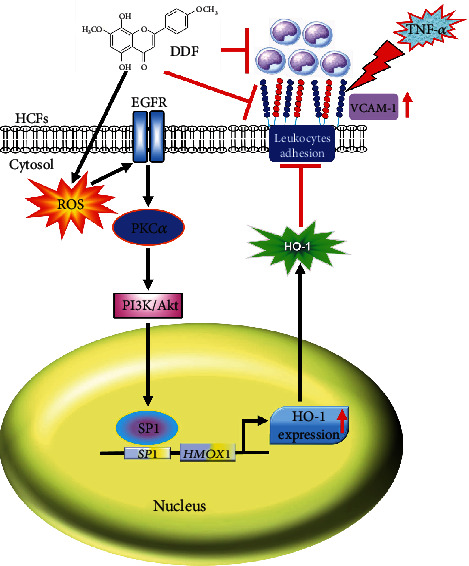
Schematic representation of signaling pathways involved in DDF-induced HO-1 expression and anti-inflammatory effects in HCFs. DDF stimulates ROS generation and activates EGFR/PKC*α*/PI3K/Akt cascade-dependent Sp1 activity, leading to binding with HO-1 promoter and induction of HO-1 expression. Moreover, DDF-mediated HO-1 expression and DDF itself attenuated TNF-*α*-activated intracellular signal expression, which can diminish TNF-*α*-induced VCAM-1 expression in HCFs.

HO-1 can act as an anti-inflammatory and antioxidant molecule that exerts cardioprotective effects and ameliorates oxidative stress, fibrosis, and hypertrophy [29]. Flavonoids are found in medicinal herbs, tea, and fruits. They belong to a family of polyphenols. Recently, we elucidated that DDF upregulates the expression of HO-1 through ROS/p38 MAPK-dependent Nrf2 activation in HCFs [12]. Here, we observed that DDF alternatively stimulates the generation of ROS linked to the EGFR/PKC*α*/PI3K/Akt pathway. This action leads to the interaction of Sp1 with the promoter of HO-1 and results in upregulation of HO-1 protein in HCFs (Figure 6). We also demonstrated that the TNF-*α*-mediated adhesion of THP-1 monocytes associated with expression of VCAM-1 protein could be protected by increased protein expression of HO-1 generated by DDF, which was rescued by ZnPP IX, one inhibitor of HO-1. Our findings indicate that DDF is a potential HO-1 inducer, which protects against inflammatory diseases.

NADPH oxidase- (NOX-) derived ROS can act as second messengers and activate their downstream signaling components such as EGFR, which has been revealed in various cell models to promote upregulation of HO-1 level [19, 20, 26]. Studies have demonstrated that butein and phloretin protect against oxidative stress by induction of GSH synthesis and expression of HO-1 protein [30]. Previously, we showed that in the alveolar epithelial cells of humans, rosiglitazone upregulates expression of HO-1 through a pathway dependent on NOx/ROS which suppresses lipopolysaccharide-mediated lung inflammation [31]. Recently, we revealed that DDF-stimulated generation of ROS and expression of HO-1 were inhibited by the N-acetylcysteine, a ROS scavenger, or GSH pretreatment, but not by the NOx inhibitor diphenyleneiodonium chloride or mitochondrial ROS scavenger MitoTempo, in HCFs [12]. Those findings suggested that in HCFs, DDF-stimulated ROS generation resulted from an imbalance between GSH-disulfide and GSH, which further induced expression of HO-1 protein dependent on the pathway activation of p38 MAPK/Nrf2 [12]. Here, the mechanisms of DDF-induced expression of HO-1 were expanded by results showing ROS-dependent activation of EGFR (a receptor tyrosine kinase) to be involved in DDF-induced responses. These results were verified by pretreatment with an EGFR inhibitor, AG1478, which attenuated DDF-mediated EGFR phosphorylation and upregulation of HO-1 expression. Furthermore, the roles of ROS in DDF-stimulated responses were ascertained by GSH pretreatment, which inhibited DDF-stimulated EGFR phosphorylation, suggesting that ROS-dependent EGFR activation upregulates the expression of HO-1 generated by DDF. The above results are compatible with the studies revealing ROS to activate its downstream signaling components EGFR or PDGFR, which leads to increased expression of HO-1 in many cell types [19, 20, 26]. Our present study suggests that increased ROS levels could activate EGFR-dependent upregulated expression of HO-1 in HCFs.

PKCs possess several cellular physiological and pathological functions, such as regulating cell growth, mediating the immune response, and regulating transcription. PKCs achieve these functions by initiating the phosphorylation of other signaling components. Kim et al. [14] revealed that PKC is a modulator of HO-1 expression. Qin et al. [32] reported that sinomenine (which is derived from medicinal herbs and is used to treat rheumatoid diseases) in HEK293 cells can activate various signaling kinases (e.g., PKC) to induce HO-1 protein expression. Here, our results in HCFs demonstrated that a ROS/EGFR pathway activates PKCs, thereby leading to upregulated expression of HO-1. The concept that PKC*α* is involved in HO-1 induction was supported by pretreatment with a PKC*α* inhibitor, Gö6976, which inhibited the HO-1 expression associated with DDF-mediated PKC*α* phosphorylation. DDF-stimulated PKC*α* phosphorylation was mitigated by AG1478 pretreatment, but not by LY294002 pretreatment. These findings suggest that PKC*α* activation mediated through a ROS-dependent EGFR pathway participates in the DDF-induced upregulated expression of HO-1.

Sun et al. [33] demonstrated that niacin stimulates a signaling cascade of PI3K/Akt via PDGFR/EGFR and PKC pathways in A431 cells. PI3K/Akt possesses a crucial effect on physiological functions of cells and several disorders, including cancer, cardiac hypertrophy, and heart failure [34, 35]. In various cell models, PI3K/Akt is the downstream component of EGFR and can induce upregulation of several genes (e.g., *HO-1*) [20, 23, 24]. In various cell types, PI3K/Akt can be activated by EGFR and PKCs [33], which participate in the expression of HO-1 induced by various stimuli [25, 31]. About the present study, PI3K/Akt participated in the DDF-induced increased protein level of HO-1, which was inhibited by Akt siRNA transfection or pretreatment with the PI3K inhibitor LY294002. We also found that Akt phosphorylation triggered by DDF was needed for upregulated expression of HO-1, which was mitigated by pretreating cells with AG1478 or Gö6976, therefore suggesting that EFGR and PKC*α* are the upstream components of PI3K/Akt in HCFs. These findings are compatible with observations in PC12 cells demonstrating that carnosol mediates through a PI3K/Akt pathway to induce HO-1 production [36]. In addition, glycyrrhizin can upregulate HO-1 expression via Akt phosphorylation, which protects against sodium iodate-induced ROS and apoptosis in retinal pigment epithelia [37]. About the present study, induction of HO-1 by DDF was reliant on an EGFR/PKC*α* signaling pathway to activate PI3K/Akt in HCFs.

The transcription factor Sp1 belongs to the Sp/Krüppel-like factor (KLF) family, which has been reported to regulate gene transcription by binding directly to DNA with its zinc finger protein motif. Multiple regulatory regions within *HO-1* have been revealed to interact with transcription factors such as Sp1 [38]. Thus, Sp1 is implicated in upregulated expression of HO-1 induced by various stimuli. Previously, we showed that in RBA-1 cells, Sp1 is recruited to the HO-1 promoter region and binds to the binding sites of ARE upon challenge with CORM-2 [26]. Gómez-Villafuertes et al. [39] indicated that EGFR activation is associated with the PI3K/Akt pathway, which triggers Sp1 phosphorylation. Rojo et al. [40] demonstrated that PI3K/PKC*ζ* increases Sp1 phosphorylation and upregulates HO-1 levels. Lee et al. [41] indicated that Sp1 is activated by the redox state. Besides, PKCs have been reported to be involved in Sp1 phosphorylation [42]. Consistently, we found that Sp1 phosphorylation participated in the increased expression of HO-1 generated by DDF, decreased by the Sp1 inhibitor mithramycin A or Sp1 siRNA. Furthermore, we found that Sp1 phosphorylation could enhance its interaction with the binding site of ARE in the promoter of HO-1, which was blocked by the inhibitor of EGFR, PI3K, PKC*α*, or ROS scavenger GSH. Therefore, we concluded that DDF-induced increased expression of HO-1 is mediated through Sp1 activity in HCFs.

TNF-*α* is a major cytokine that promotes inflammation. It participates in the pathogenesis of cardiovascular diseases. The level of TNF-*α* can be upregulated by many types of cardiac cells, including cardiac fibroblasts. In the latter, TNF-*α* can enhance matrix metalloproteinase secretion and stimulate cell proliferation, fibronectin deposition, and transdifferentiation of fibroblasts into myofibroblasts [43]. Previously, we revealed that TNF-*α* promoted monocytes to adhere to HCFs resulting from upregulated expression of VCAM-1 [8, 27]. Adhesion molecules have crucial roles in cardiac remodeling because they modulate the rolling and adhesion of inflammatory immune cells into tissues. A growing body of evidence indicates that agents that can inhibit expression of adhesion molecules could be beneficial for the management of cardiac diseases [43–46]. Here, we demonstrated that DDF, through HO-1 upregulation, decreased the expression of VCAM-1 stimulated by TNF-*α*, which led to inhibition of adhesion of monocytes to HCFs. The inhibitory effect of DDF on monocyte adhesion was reversed by ZnPP IX (an inhibitor of HO-1) pretreatment. Hence, the DDF's effects on inhibiting TNF-*α*-mediated inflammatory responses could be dependent upon HO-1 induction.

## 5. Conclusions

We expanded our recent findings [12] and demonstrated that an alternative pathway in HCFs, through activation of ROS-dependent EGFR/PKC*α*/PI3K/Akt/Sp1, also is involved in the upregulated expression of HO-1 enhanced by DDF. Moreover, our discoveries suggested that DDF-induced upregulated expression of HO-1 could inhibit the VCAM-1 induction associated with adhesion of inflammatory cells to HCFs stimulated with TNF-*α*. Thus, DDF may be a strategy in the management of cardiac inflammation. Further exploration of the effects of DDF on inflammation in *in vivo* models of cardiovascular inflammatory diseases is warranted.

## Data Availability

The data used to support the findings of this study are available from the corresponding author upon request.

## Abbreviations

ARE: Antioxidant response element
ChIP: Chromatin immunoprecipitation
DMEM/F-12: Dulbecco's modified Eagle's medium/Ham's F-12
DPI: Diphenylene iodonium chloride
ECL: Enhanced chemiluminescence
ECM: Extracellular matrix
EGFR: Epidermal growth factor receptor
ERK: Extracellular regulated protein kinase
FBS: Fetal bovine serum
GAPDH: Glyceraldehyde-3-phosphate dehydrogenase
HO-1: Heme oxygenase-1
HCF: Human cardiac fibroblast
MAPK: Mitogen-activated protein kinase
NAC: *N*-Acetyl-L-cysteine
Nrf2: NF-E2-related factor 2
PDGFR: Platelet-derived growth factor receptor
PI3K: Phosphatidylinositol 3-kinase
PKC: Protein kinase C
PMSF: Phenylmethylsulfonyl fluoride
ROS: Reactive oxygen species
PCR: polymerase chain reaction
SiRNA: Small interfering RNA
Sp1: Specificity protein 1
TNF-*α*: Tumor necrosis factor-alpha
TTBS: Tween-Tris-buffered saline
VCAM-1: Vascular cell adhesion molecule-1.
